# Gut microbiota of the threatened takahē: biogeographic patterns and conservation implications

**DOI:** 10.1186/s42523-021-00158-5

**Published:** 2022-01-25

**Authors:** Annie G. West, Anne DeLaunay, Phil Marsh, Elena K. Perry, Megan Jolly, Brett D. Gartrell, An Pas, Andrew Digby, Michael W. Taylor

**Affiliations:** 1 School of Biological Sciences, University of Auckland, Private Bag 92019, Auckland, 1142 New Zealand; 2 Takahē Recovery Programme, Department of Conservation, Lakefront Drive, Te Anau, New Zealand; 3 School of Veterinary Science, Massey University, Palmerston North, New Zealand; 4 New Zealand Centre for Conservation Medicine, Auckland Zoo, Auckland, New Zealand; 5 Present Address: Division of Biology and Biological Engineering, California Institute of Technology, Pasadena, CA USA

**Keywords:** Microbiome, Endangered, Bird, Avian, Conservation, Microbiota

## Abstract

**Background:**

The Aotearoa New Zealand takahē (*Porphyrio hochstetteri*), once thought to be extinct, is a nationally threatened flightless rail under intensive conservation management. While there has been previous research into disease-related microbes in takahē, little is known about the microbes present in the gastrointestinal tract. Given the importance of gut-associated microbes to herbivore nutrition and immunity, knowledge of these communities is likely to be of considerable conservation value. Here we examined the gut microbiotas of 57 takahē at eight separate locations across Aotearoa New Zealand.

**Results:**

Faecal samples, taken as a proxy for the hindgut bacterial community, were subjected to 16S rRNA gene amplicon sequencing using Illumina MiSeq. Phylogenetic analysis of > 2200 amplicon sequence variants (ASVs) revealed nine main bacterial phyla (*Acidobacteriota, Actinobacteriota*, *Bacteroidota*, *Campilobacterota, Firmicutes*, *Fusobacteriota*, *Planctomycetota, Proteobacteria*, and *Verrucomicrobiota*) that accounted for the majority of sequence reads. Location was a significant effect (*p* value < 0.001, 9999 permutations) that accounted for 32% of the observed microbiota variation. One ASV, classified as *Lactobacillus aviarius*, was present in all samples at an average relative abundance of 17% (SD = 23.20). There was strong evidence (*p* = 0.002) for a difference in the abundance of the genus *Lactobacillus* between locations. A common commensal bacterium previously described in takahē, *Campylobacter* spp., was also detected in most faecal samples.

**Conclusions:**

Location plays a pivotal role in the observed variation among takahē gut bacterial communities and is potentially due to factors such as supplemental feeding and medical treatment experienced by birds housed in captivity at one of the eight sampled sites. These data present a first glimpse of the previously unexplored takahē gut microbiota and provide a baseline for future microbiological studies and conservation efforts.

**Supplementary Information:**

The online version contains supplementary material available at 10.1186/s42523-021-00158-5.

## Introduction

The takahē (*Porphyrio hochstetteri*) is a threatened, flightless species of rail endemic to Aotearoa New Zealand. After being declared extinct in 1898, a small population was rediscovered in a remote mountain range of the southern Te Waipounamu South Island 50 years later. Takahē, the largest member of the Rallidae family, were once widespread across numerous edge-type habitats, but have since taken refuge in alpine grass habitats due to climate change, habitat loss, and the arrival of humans and introduced mammalian predators [12, 25, 40, 65]. Population numbers have fluctuated since rediscovery due to predation by stoats and competition with deer for food [28, 40, 45, 76]. Several breeding programmes and insurance populations were therefore established to safeguard against drastic reductions in takahē numbers [13]. After decades of dedicated conservation efforts the population has now reached > 400 individuals and additional suitable habitats are being identified to house the growing number of takahē. There are currently well-established research initiatives regarding takahē nutrition, health and breeding, and genome sequencing. However, an important aspect of takahē biology that remains understudied is the role of symbiont microbiota in takahē gut health and immunity.

The key roles of gut microbes in host digestion, nutrition and immunity are now widely recognised (reviewed by Spor et al. [61], Hall et al. [26] and Gilbert et al. [17]). Although birds constitute the largest class of tetrapods, avian microbiota studies remain largely outnumbered by those of the mammalian microbiota. Here, we use the term ‘microbiota’ to refer to a community of microorganisms present in a defined environment (e.g. the gut), while the term ‘microbiome’ encompasses the microbiota as well as their theatre of activity, i.e. genomic structures and elements, metabolites and surrounding environmental conditions [7, 38]. Some existing avian-related studies have focused on digestive capabilities, such as in the foregut-fermenting hoatzin [19, 20], or were conducted within the scope of conservation management programmes, including the critically endangered kākāpō (*Strigops habroptilus*) [49, 67], northern bald ibis (*Geronticus eremita*) [60] and two Old World vultures, the Griffon (*Gyps fulvus*) and Egyptian vulture (*Neophron percnopterus*) [5]. However, most studies of the avian microbiota are concentrated on commercially farmed species (e.g. broiler chicken *Gallus gallus domesticus*, turkey *Meleagris gallopavo domesticus*, ostrich *Struthio camelus*) and aim to understand how beneficial microbes may influence animal growth, fitness, and reproduction [24, 68, 70]. While the exact relationship between the microbiota and host fitness remains unclear, gut microbes are indeed essential to the digestion of complex plant polysaccharides and to host resilience against invading pathogens. Herbivores rely on microorganisms in the gut to break down fibrous plant material and convert these complex molecules to more easily digestible short-chain fatty acids (SCFA) [30, 37].

Takahē are near-exclusive herbivores that feed predominantly on *Chionochloa* tussock meristems, tikumu mountain daisy (*Celmisia petrieiv*) and huarau fern (*Hypolepis millefolium*) rhizomes in their native habitat, supplemented by the occasional invertebrate [, 34, 47, 54, 79] and producing a staggering 9 m of largely undigested, grassy stool each day [27, 34, 54]. Takahē likely support a form of fermentation in a pair of slightly elongated, simple caeca where some plant matter is diverted, but this represents a very small proportion of the ingested material [27, 62]. Dietary habits are likely to differ significantly among takahē populations, especially in sanctuaries that incorporate supplemental feeding schemes to support breeding pairs (all locations that provide supplementary feed use the same cereal-based pellet).

Significant changes in gut microbiota composition have been observed for threatened species housed in captive environments and/or undergoing medical treatment [42, 74]. The potential for captive animals to lose key components of the microbiota that are essential to digestion and host function in the wild is concerning and could in some cases result simply from insufficient variation in the diet. Loss of key microbial players could impact nutritional efficiency and functional capacity of the gut microbiota, particularly in herbivorous animals where some microbes play important roles in degrading toxic plant compounds (xenobiotics) [1, 2, 9, 16]. Changes to the gut microbiota that impact host health could also lower the efficacy of breeding and translocation programmes.

Given the importance of the gut microbiota to host health, particularly in herbivore digestion and nutrition, baseline knowledge of key microbial taxa in the takahē gut microbiota would add greatly to our knowledge of takahē biology. Existing microbiology-based research in takahē has focused solely on specific microbial taxa such as *Campylobacter* [22, 23], which has been identified as a common commensal bacterium in takahē faecal material, and the potential for pathogen transmission among sub-populations with regular translocations [21]. The primary aim of this present study was to characterise the bacterial community colonising the gut of adult takahē from different geographic locations using faecal samples as a proxy for gut microbial assemblages [66]. It represents an initial investigation into the takahē gut microbiota that should ultimately facilitate finer-scale studies which aid in managing the health and conservation of this rare, enigmatic bird.

## Materials and methods

### Sample collection

Fresh or recently deposited (< 1 h) faecal material collected from 57 adult takahē (representing ~ 14% of the global population at the time of sampling) between August 2016 and June 2017 (see Additional file 1) by MJ and New Zealand Department of Conservation (Te Papa Atawhai; NZDOC) staff was placed directly into 15 mL sterile polypropylene tubes containing RNA*later* (for ease of transport and use in remote field locations; [59]), then stored overnight at 4 °C followed by subsequent storage at -20 °C. The faecal material was obtained from takahē residing in sanctuary sites and from the sole wild population at the time of sampling, totalling eight separate sampling locations across Aotearoa New Zealand: Burwood Takahē Centre (Te Anau), Cape Sanctuary (Te Kauwae-a-Māui Peninsula, 39° 38′ S, 177° 05′ E), Mana Island (Kapiti Coast, 41° 05′ S, 174° 46′ E), Motutapu Island (Hauraki Gulf, 36° 45′ S, 174° 54′ E), Te Puhi-a-Noa Murchison Mountain Range (wild population; “Murchison Mountains”, 45° 16′ S, 167° 32′ E), Rotoroa Island (Hauraki Gulf, 36° 49′ S, 175° 12′ E), an island in the Foveaux Strait (Te Ara-a-Kewa), and Tiritiri Matangi Island (Hauraki Gulf, 36° 36′ S, 174° 53′ E). Samples were shipped to the Waipapa Taumata Rau University of Auckland on ice for DNA extraction and sequencing and were stored on arrival at − 20 °C. Metadata for this study included takahē age, sex, supplemental feeding regime, puppet vs wild rearing, location, nest site, hatch site, and habitat type (regenerating vs established) (metadata table included as Additional file 1). Burwood Takahē Centre houses a captive breeding population with intensive anthropogenic management, while the other subpopulations at Cape Sanctuary, Rotoroa Island, Mana Island, Tiritiri Matangi Island, Motutapu Island, and the Foveaux Strait island represent insurance populations that also experience regular anthropogenic management. The wild, free-to-roam population is largely left alone with little to no anthropogenic interference. Supplementary feed consists of a cereal-based pelleted ration that is the same for all locations. Locations where takahē are regularly provided supplementary feed (at least once per week) include Burwood Takahē Centre, Cape Sanctuary, Rotoroa Island, Mana Island and Tiritiri Matangi Island. Occasional supplementary feeding (once per fortnight or less) is provided at Motutapu Island, while no supplemental feed is provided to the wild Te Puhi-a-Noa Murchison Mountain population or to the takahē on the Foveaux Strait island.

Faecal samples are often collected as part of routine management of takahē. Samples collected for this study were approved by NZDOC and did not require ethics approval from NZDOC Animal Ethics Committee, which upholds NZDOC’s obligations under the New Zealand Animal Welfare Act.

### DNA extraction, PCR and 16S rRNA gene sequencing

Total DNA was extracted from thawed samples using a bead-beating method previously described by Perry et al. [49]. In brief, 100 mg of faecal material was washed twice with 70% ethanol before suspension in a high-salt, CTAB-based extraction buffer, followed by agitation in a FastPrep FP120 bead beater at 5.5 ms^−1^ for 30 s. Samples were then repeatedly incubated at 65 °C, cleaned with a 24:1 chloroform/isoamyl alcohol mix and incubated overnight twice at -20 °C with 0.6 vol isopropanol and 0.1 vol sodium acetate (3 M, pH 5.2), followed by two rounds of ethanol wash. The final pellet was suspended in 20 µL of 10 mM Tris–HCl (pH 8). The variable V3-V4 region of the 16S rRNA gene was amplified following Perry et al. [49], with PCR reactions performed in triplicate for each faecal sample then pooled to increase DNA yield. A KAPA 3G Plant PCR kit was used for amplification with initial denaturation at 95 °C for 3 min, followed by 35 cycles of denaturation at 95 °C for 20 s, annealing at 57 °C for 15 s, and extension at 72 °C for 30 s. The final elongation was set at 72 °C for 1 min. Amplicon size and the absence of a band for negative controls (including laboratory extraction controls and PCR amplification no-template controls) were verified on a 1% agarose gel with SYBR Safe DNA Gel Stain (Invitrogen, New Zealand). PCR products were purified using AMPure XP (Beckman Coulter, New Zealand) beads and DNA concentration was quantified on a Qubit Fluorometer 1.0 (Invitrogen, New Zealand) using the High Sensitivity dsDNA kit. Samples were normalised to 10 ng/µL for library preparation and sequencing by New Zealand Genomics Ltd on an Illumina MiSeq with 2 × 300 bp chemistry.

### Sequence data analysis

All raw paired-read 16S rRNA gene sequence data were analysed on the New Zealand eScience Infrastructure (NeSI) High Performance Computing cluster Mahuika in R (version 4.0.1 [51]) using the DADA2 software package (version 1.16 [10]). Primer regions were removed and forward and reverse reads subsequently truncated to 280 bp and 240 bp, respectively. Sequence reads shorter than the truncated value were discarded, as were reads where truncQ < 2 or where the number of expected errors exceeded 3 for forward and reverse reads (–maxEE parameter). The DADA2 error learning algorithm was then applied to the forward and reverse reads, which were subsequently dereplicated into unique sequences. The DADA2 core sample algorithm was applied to the dereplicated sequences, which were merged thereafter to obtain the full denoised sequence. Sequence chimeras were excluded and taxonomy assigned using the SILVA species version 138 ribosomal RNA reference database [50] (see Additional file 2 for taxonomic assignments of ASVs). A phylogenetic tree was constructed using the DECIPHER (version 2.16.1 [80]) and phangorn (version 2.5.5 [58]) packages. Sequence files and metadata for all samples were uploaded to SRA under Bioproject number PRJNA737580.

The resulting ASV table (see Additional file 3 for non-rarefied ASV table), taxonomic assignments and phylogenetic tree were combined with corresponding metadata to construct a phyloseq object using the R (version 4.1.0 [52]) package phyloseq (version 1.34.0 [43]). Non-target sequences including chloroplasts and mitochondria were removed from the data set using phyloseq’s prune_taxa function. The ASV table was also filtered to remove low-abundance ASVs (total relative abundance < 0.001%) and ASVs not assigned to phylum level. The dataset was then rarefied to the minimum number of reads per sample (8000) to account for substantial differences in library size for subsequent analyses [73]. ASVs are numbered in decreasing order of their relative sequence abundance across the dataset.

To explore variation among bacterial communities grouped by significant covariates (beta-diversity), generalised UniFrac (GUniFrac version 1.1 [11]) and Bray–Curtis (vegan) distances were calculated from the rarefied data and subjected to non-metric multidimensional scaling (nMDS) and principal coordinate analysis (PCoA) ordination (see Additional file 5: Figure S1 for gUniFrac PCoA visualisations). We tested for significant associations between dissimilarity matrices and corresponding covariates using the PERMANOVA adonis2 function of the vegan package with 9999 permutations. Significant PERMANOVA models were further subjected to pairwise comparison testing using the pairwise.adonis function of the pairwiseAdonis package (version 0.4 [39]). We subsequently used the vegan functions betadisper and permutest to test for homogeneous group dispersion.

Alpha-diversity indices were explored by applying phyloseq’s estimate_richness function to the rarefied data. Associations between alpha-diversity indices and corresponding metadata (age, sex, supplemental feeding, rearing method, location, and habitat type) were tested using Kruskal–Wallis or ANOVA tests (dependent on Shapiro Wilks normality tests) using the vegan package (version 2.5–7 [48]). Post-hoc pairwise comparisons were performed for significant Kruskal–Wallis and ANOVA models using Dunn’s test (dunn.test package version 1.3.5 [14]) with Benjamini–Hochberg *p* value correction [6] or Tukey’s HSD test (stats package version 4.1.0 [52]). The core microbiota was identified as those ASVs present in 70% of individuals at > 0.01% relative abundance and genera present in 75% of individuals at > 1% relative abundance using the package microbiome (version 1.13.9 [33]). The relative distribution of core genera within each location was plotted with associated Kruskal–Wallis tests for each taxon using a modified version of a script published on GitHub by Bodkhe et al. [8].

We then created a separate taxonomic level which concatenated genus-and species-level assignments together and agglomerated the phyloseq object to this taxonomy group. The plyr package (version 1.8.6 [75]) was leveraged to group less abundant ASVs into the category ‘Others’ based on a per-species mean relative abundance of < 0.3% and the data were plotted against location for all faecal samples. Finally, the DESeq2 package (version 1.30.0 [36]) was used to test for differential abundance of ASVs (test = “Wald”, fitType = “local”) between Te Puhi-a-Noa Murchison Mountain and Burwood Takahē Centre samples using the non-rarefied data (as described in [8]).

All data were visualised using the R packages ggplot2 (version 3.3.3 [77]), ggpubr (version 0.4.0 [31]), cowplot (version 1.1.1 [78]), ggsci (futurama palette, version 2.9 [81]) and Manu (‘takahē’ palette specifically designed on the colours of the takahē, version 0.0.1 [53, 63]).

The markdown file for all analyses is included as Additional file 4.

## Results

In total, 7,877,544 raw 16S rRNA gene sequence reads were obtained from the 57 takahē faecal samples, with 1,798,118 merged reads remaining after quality and chimera filtering. Following removal of non-target sequences and low-abundance ASVs, 2,224 unique ASVs were identified across the entire data set. The ASV table was rarefied to 8000 reads per sample for statistical analyses, which reduced the total number of ASVs to 2,222. The number of ASVs per sample ranged from 86 to 555, with an average of 287. Overall, 99.99% of filtered sequence reads could be assigned to at least phylum level.

**Fig. 1:**
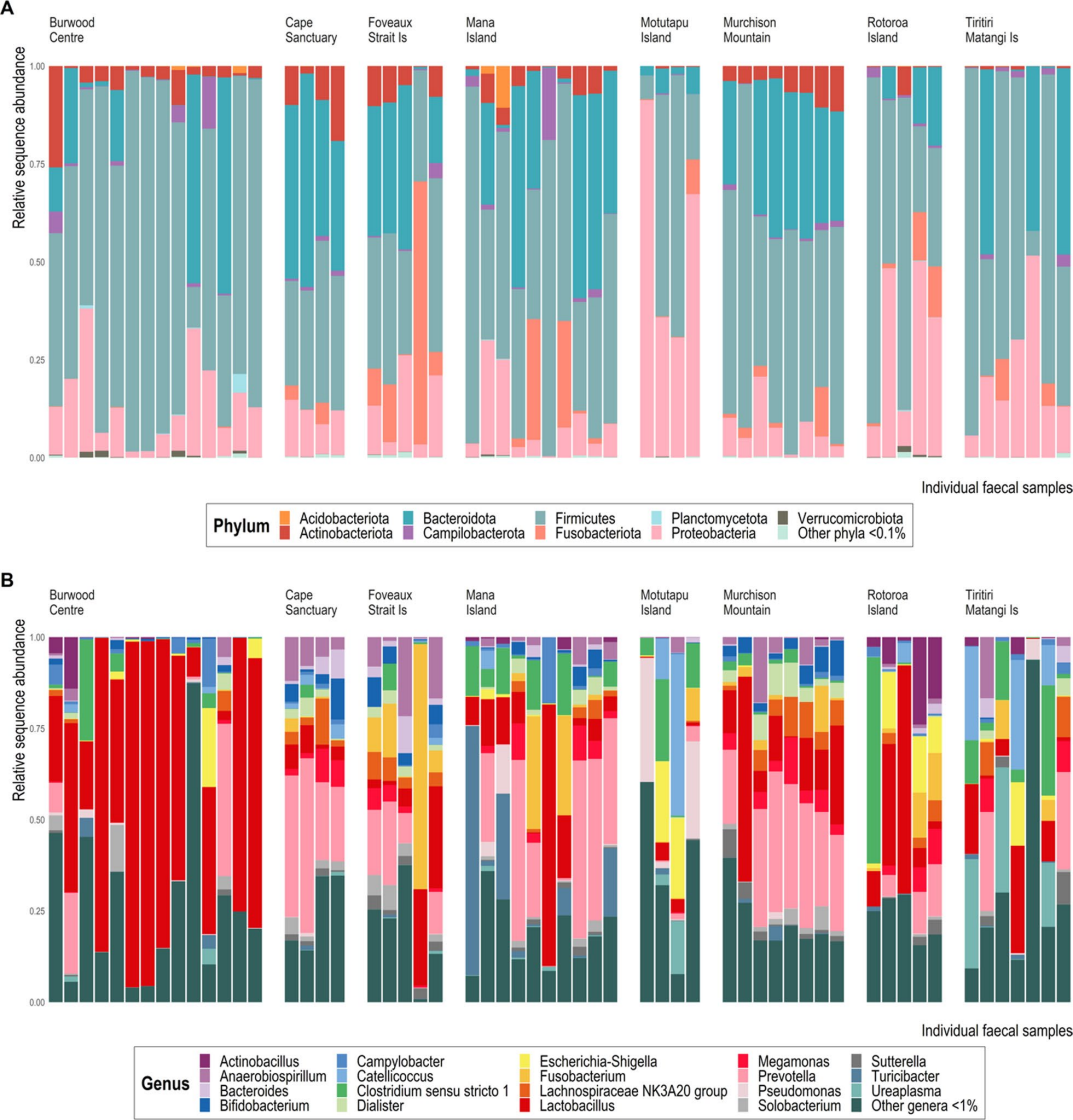
16S rRNA gene-based taxonomic distribution of bacteria within takahē faecal samples at **A** phylum and **B** genus levels. Taxa with mean relative 16S rRNA gene sequence abundance < 0.1% and < 1%, respectively, are grouped together as ‘Others’. Each bar represents a single takahē faecal sample. Wild population = Te Puhi-a-Noa Murchison Mountain, captive population = Burwood Takahē Centre, insurance populations = Cape Sanctuary, Foveaux Strait island, Mana Island, Motutapu Island, Rotoroa Island and Tiritiri Matangi Island. No supplementary feed was provided to Te Puhi-a-Noa Murchison Mountain or Foveaux Strait Is takahē

Nine bacterial phyla each exceeded 0.1% mean relative sequence abundance (MRA) across the entire data set, namely *Acidobacteriota* (MRA = 2.90e^−3^ ± SD 0.02)*, Actinobacteriota* (MRA = 0.04 ± 0.05), *Bacteroidota* (MRA = 0.20 ± 0.20), *Campilobacterota* (MRA = 0.01 ± 0.03)*, Firmicutes* (MRA = 0.52 ± 0.25), *Fusobacteriota* (MRA = 0.04 ± 0.11), *Planctomycetota* (MRA = 1.35e^−3^ ± 6.41e^−3^)*, Proteobacteria* (MRA = 0.17 ± 0.18), and *Verrucomicrobiota* (MRA = 1.78e^−3^ ± 4.02e^−3^) (Fig. 1A). Relative sequence abundances varied considerably among individual birds and locations. *Firmicutes* dominated the data set, accounting for 51.9% of rarefied reads. *Bacteroidota* and *Proteobacteria* accounted for the majority of remaining reads (19.98% and 17.2% respectively), while *Acidobacteriota, Actinobacteriota*, *Campilobacterota, Fusobacteriota, Planctomycetota* and *Verrucomicrobiota* together comprised 10.6% of total sequence reads. The majority of rarefied and filtered ASVs were assigned a genus classification (96.5%).

Within the *Firmicutes* phylum, *Lactobacillus* was by far the most numerous genus (Fig. 1B), followed by *Clostridium *sensu stricto* 1, Turicibacter* and *Catellicoccus*. Indeed, *Lactobacillus* was the most abundant bacterial genus overall, representing 23.6% of sequences that were assigned at genus level. *Prevotella* was the most abundant genus of the *Bacteroidota* phylum and the second most abundant taxon overall (14.3% of all sequences assigned to genus). For *Proteobacteria*, the three most abundant genera were *Anaerobiospirillum*, *Escherichia-Shigella*, and *Pseudomonas*.

**Fig. 2:**
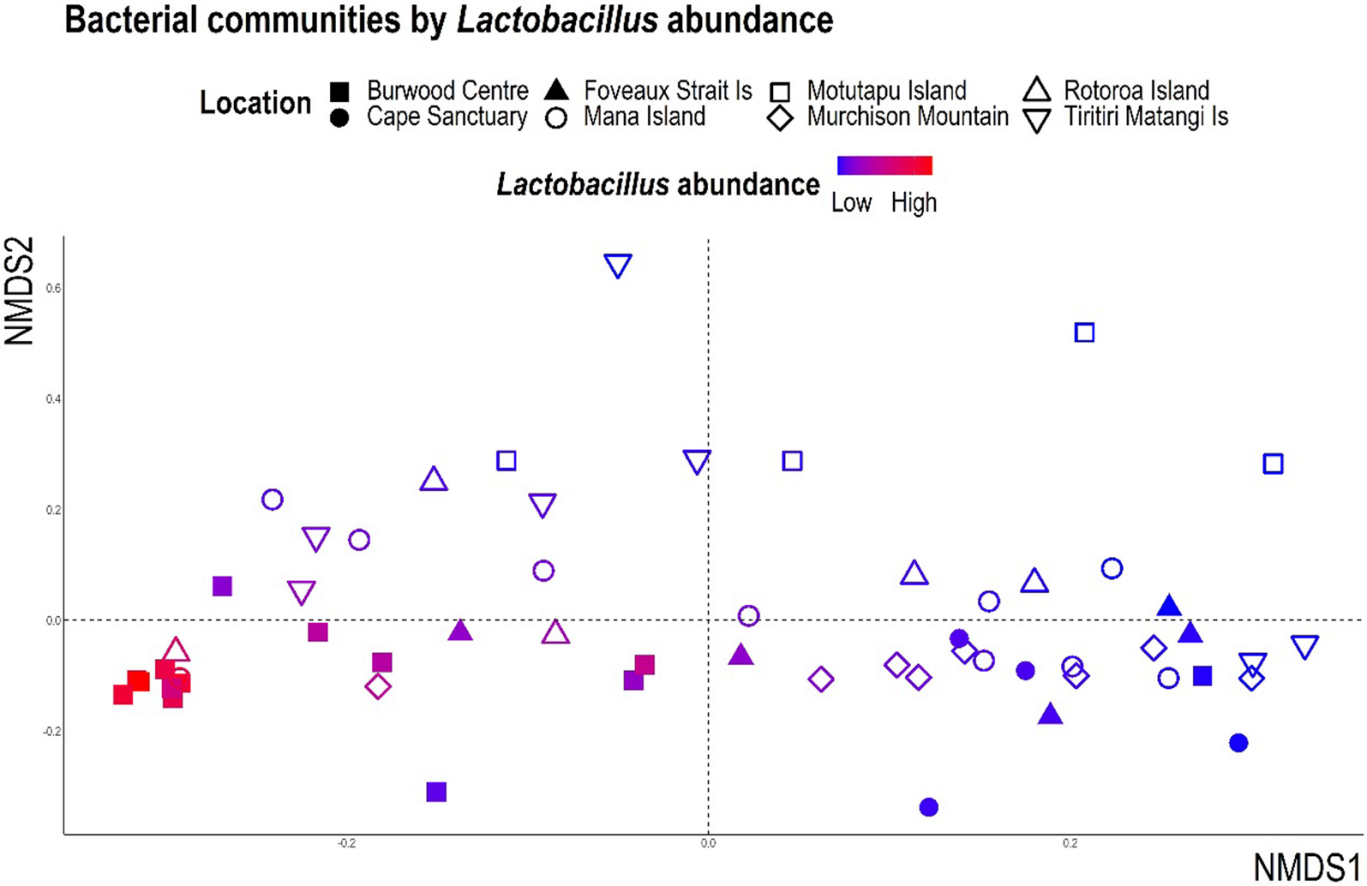
Bray–Curtis dissimilarity distances visualised via NMDS ordination. Bacterial communities are coloured according to relative Lactobacillus 16S rRNA gene sequence abundance and shaped according to location. Each dot of the NMDS represents a single takahē faecal sample (MDS stress = 0.17)

We observed a significant dispersion of bacterial communities by location (*p* value < 0.001; Table 1) with Bray–Curtis NMDS and gUniFrac PCoA ordinations (Fig. 2 and Additional file 5: Figure S1, respectively). Overall, location accounted for 32% of the observed variation in faecal bacterial communities; this proportion dropped to 20% when accounting for differences in supplemental feeding among sites. The first ordination axis separates the takahē faecal communities from Burwood Takahē Centre from those in the Te Puhi-a-Noa Murchison Mountains, representing the most- and least-intensively managed takahē populations, respectively (Fig. 2 and Additional file 5: Figure S1). Separation of samples is at least partly attributable to much higher relative sequence abundances of *Lactobacillus* in the Burwood samples (Fig. 2 and Additional file 5: Figure S1). Pairwise PERMANOVA of both Bray–Curtis and gUniFrac matrices indicated that Burwood Takahē Centre samples were responsible for much of this significant variation (Additional file 5: Table S1). The betadisper test for homogeneity of multivariate dispersions was non-significant for both Bray–Curtis and gUniFrac matrices, indicating that within-group variances were all similar, i.e. all groups showed similar dispersion of samples from their centroid.

**Table 1:** Statistical outputs for beta-diversity Bray–Curtis and gUniFrac matrices using PERMANOVA analyses performed with 9999 permutations

	PERMANOVA for Bray–Curtis matrix			PERMANOVA for gUniFrac matrix		
Covariate	*p* value	F-statistic	R^2^	*p* value	F-statistic	R^2^
Location	< 0.001***	3.27	0.32	< 0.001***	3.36	0.32
Supplemental feeding	< 0.001***	3.37	0.11	< 0.001***	3.92	0.13
Habitat type	< 0.001***	4.13	0.07	0.01*	3.11	0.05
Age	0.64	0.80	0.02	0.94	0.46	0.008
Sex	0.12	1.45	0.03	0.17	1.41	0.03
Hatch site	0.06	1.23	0.21	0.15	1.19	0.21
Nest site	0.06	1.23	0.21	0.15	1.86	0.21
Origin	0.55	0.93	0.03	0.92	0.59	0.02

Significant *p* values are denoted with asterisks (*p* < 0.05 = *, *p* < 0.01 = **, *p* < 0.001 = ***)

Supplemental feeding alone was significantly associated with microbial beta-diversity (*p* value < 0.001, Table 1) that accounted for 11–13% of variation among bacterial communities but did not have homogenous group dispersion (Bray–Curtis *p* value < 0.001, F = 12.78; gUniFrac *p* value = 0.002, F = 7.57). We therefore cannot rule out that the significance of our PERMANOVA test was only due to unequal dispersion within the supplemental feeding groups and not also a result of varying bacterial composition among groups. However, after removing the "Occasional" supplemental feeding group due to small sample size (n = 4), both the betadisper and PERMANOVA results remained significant (Bray–Curtis PERMANOVA *p* value < 0.001, F = 24.21 and betadisper *p* value = 0.001, F = 4.03; gUniFrac PERMANOVA *p* value = 0.002, F = 4.71 and betadisper *p* value = 0.002, F = 12.01). All pairwise comparisons were significant, though the comparison between “None” versus “Occasional” supplemental feeding groups yielded a much greater effect size for both Bray–Curtis and gUniFrac matrices than the other two pairwise comparisons (Additional file 5: Table S1). The centroids of the "Regular" and "None" supplemental feeding groups (Additional file 5: Figure S2A) correlate with the Burwood Takahē Centre and Te Puhi-a-Noa Murchison Mountain centroids from the location-based ordinations (Fig. 2 and Additional file 5: Figure S1), suggesting supplemental feeding is likely responsible for some of the observed bacterial variation between the intensively managed Burwood Takahē Centre takahē and the wild Te Puhi-a-Noa Murchison Mountain population. Indeed, sequential PERMANOVA (distance.matrix ~ supplemental feeding + location, by = ‘term’) and marginal PERMANOVA (distance.matrix ~ supplemental feeding + location, by = ‘margin’) indicate that the variation explained by supplemental feeding is captured when testing for location alone and is thus nested inside the variation explained by location. Similarly, the variation among bacterial communities explained by habitat type (Table 1) is captured when testing for location effect, yet also has uneven group dispersion (Bray–Curtis *p* value = 0.006, F = 8.38; gUniFrac *p* value = 0.03, F = 4.68) (Additional file 5: Figure S2B). Takahē age, sex and rearing method, as well as hatch site and nest site, did not explain a significant proportion of the variation in gut bacterial community composition (significance threshold of < 0.05; Table 1).

Alpha-diversity differed significantly by takahē location with Shannon and Inverse Simpson diversity (Additional file 5: Figure S3A). Supplemental feeding and habitat type also significantly influenced bacterial community Inverse Simpson diversity and observed species richness, respectively (Additional file 5: Figures S3B and S3C). Analyses comparing individual takahē, age or sex did not yield significant differences. Significant Tukey’s HSD and Dunn's pairwise comparisons for location and supplemental feeding effects are denoted on Additional file 5: Figures S3A and S3B. There were several instances of significant pairwise comparisons by location across both Shannon and Inverse Simpson diversity indices. However, the small sample size from Cape Sanctuary is likely biasing the significance of comparisons in which this location is included. Overall, faecal samples collected from Burwood Takahē Centre exhibited the lowest bacterial richness and diversity of all eight locations. Additionally, the bacterial communities of faecal samples from takahē experiencing regular supplemental feeding had much lower Inverse Simpson diversity compared to individuals with no supplemental feeding. Faecal samples collected from regenerating habitat had significantly higher bacterial richness than samples collected from established habitats. However, this observation may be driven by the inclusion of six locations in the former compared to two locations in the latter, which includes the low diversity Burwood Takahē Centre samples.

The core microbiota was explored using several different criteria. ASV1, identified as *Lactobacillus aviarius,* was the only ASV present in all of the faecal samples. Indeed, it was the only ASV present in $\ge$ 90% of samples at a threshold of 0.1% relative sequence abundance. An NCBI nucleotide BLAST search (standard databases and optimised for highly similar sequences) of the ASV1_*Lactobacillus aviarius* sequence suggests this is a strain of *L. aviarius araffinosis* (99.30% sequence identity), a homofermentative, strict anaerobe previously reported from chicken intestines. The mean relative sequence abundance of this ASV across all samples was 17.56% (SD = 23.20), with a range of 0.01% to 82% relative sequence abundance within a given sample. Reducing the core prevalence threshold to $\ge$ 70% identified 13 ASVs as core members at a relative sequence abundance threshold of 0.01% (in order of descending abundance): ASV1_*Lactobacillus aviarius*, ASV2_*Prevotella*, ASV3_*Fusobacterium mortiferum*, ASV4_*Clostridium *sensu stricto* 1*, ASV6_*Prevotella*, ASV12_*Lactobacillus aviarius*, ASV13_*Solobacterium*, ASV16_*Lactobacillus aviarius*, ASV23_*Bifidobacterium*, ASV24_*Lactobacillus*, ASV29_*Solobacterium*, ASV36_*Lactobacillus intermedius* and ASV83_*Lactobacillus aviarius*.

**Fig. 3:**
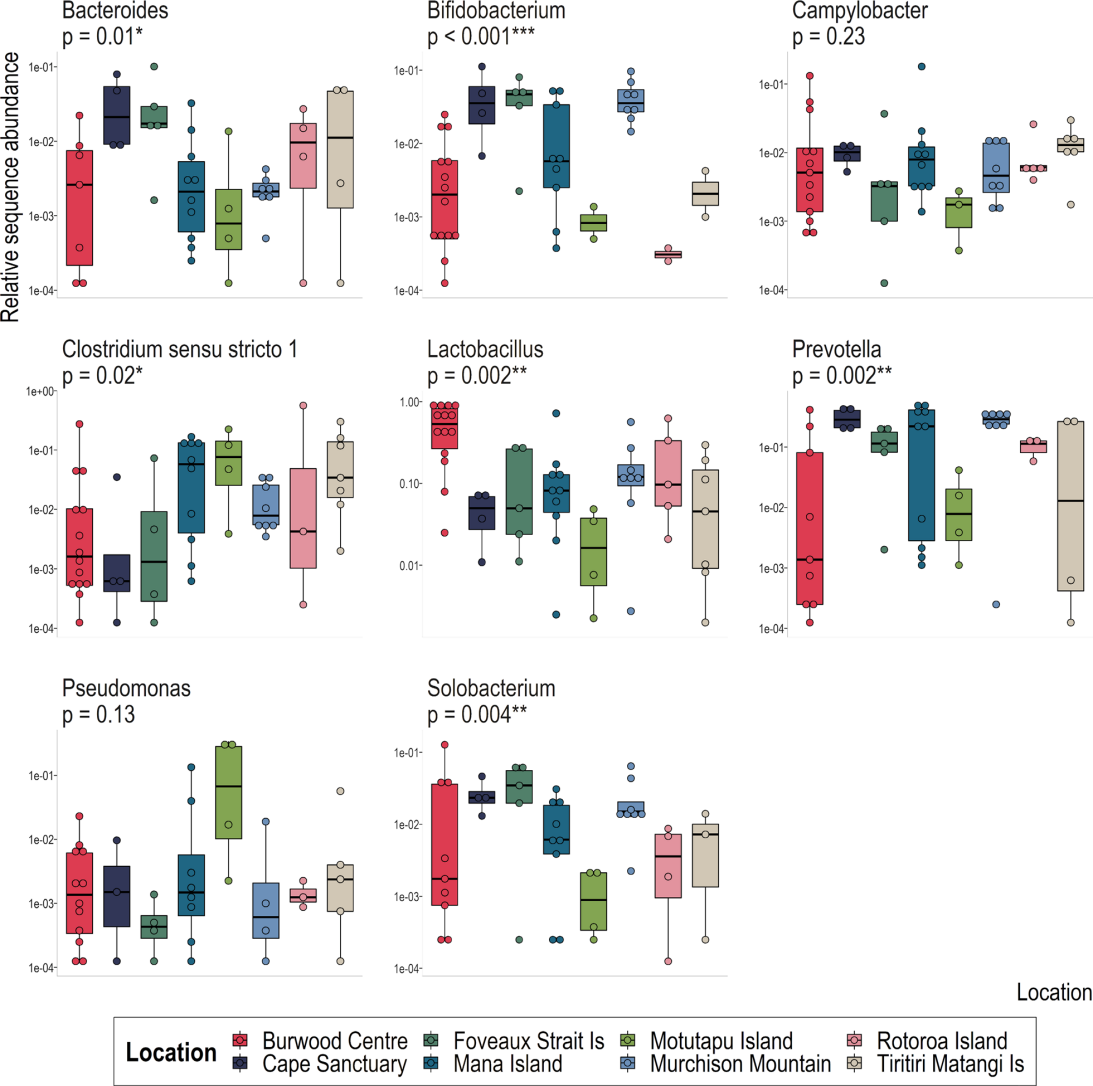
Distribution of core bacterial genera by location, with Kruskal–Wallis *p*-value displayed for each taxon. Relative 16S rRNA gene sequence abundances are log10 transformed. Significant comparisons are denoted by asterisks (*p* < 0.05 = *, *p* < 0.01 = **, *p* < 0.001 = ***)

Eight genera were identified as comprising the core gut microbiota (defined here as 1% relative abundance in $75$% of samples): *Bacteroides*, *Bifidobacterium*, *Campylobacter*, *Clostridium *sensu stricto* 1*, *Lactobacillus*, *Prevotella*, *Pseudomonas* and *Solobacterium* (Fig. 3). All genera varied considerably in their relative sequence abundances, both within and between location groupings (Fig. 3). *Campylobacter* and *Pseudomonas* were the only genera where relative sequence abundance did not differ significantly between locations. The relative sequence abundances of most core genera varied substantially among Burwood Takahē Centre samples, although the relative sequence abundance of *Lactobacillus* in Burwood Takahē Centre takahē was much greater than was observed in samples from any other location. On the other hand, *Prevotella* relative sequence abundance was significantly higher in samples from Cape Sanctuary, Foveaux Strait Island, Te Puhi-a-Noa Murchison Mountain and Rotoroa Island compared to those from Burwood Takahē Centre, Mana Island, Motutapu Island and Tiritiri Matangi Island. The relative sequence abundances of *Bifidobacterium* and *Solobacterium* were also significantly greater in Cape Sanctuary, Foveaux Strait Island, Mana Island and Te Puhi-a-Noa Murchison Mountain samples, while samples from Motutapu Island had significantly greater relative sequence abundance of *Pseudomonas* than any other location. Burwood Takahē Centre and Te Puhi-a-Noa Murchison Mountain samples were then isolated to test for differences between captive and wild gut communities at the ASV level.

**Fig. 4:**
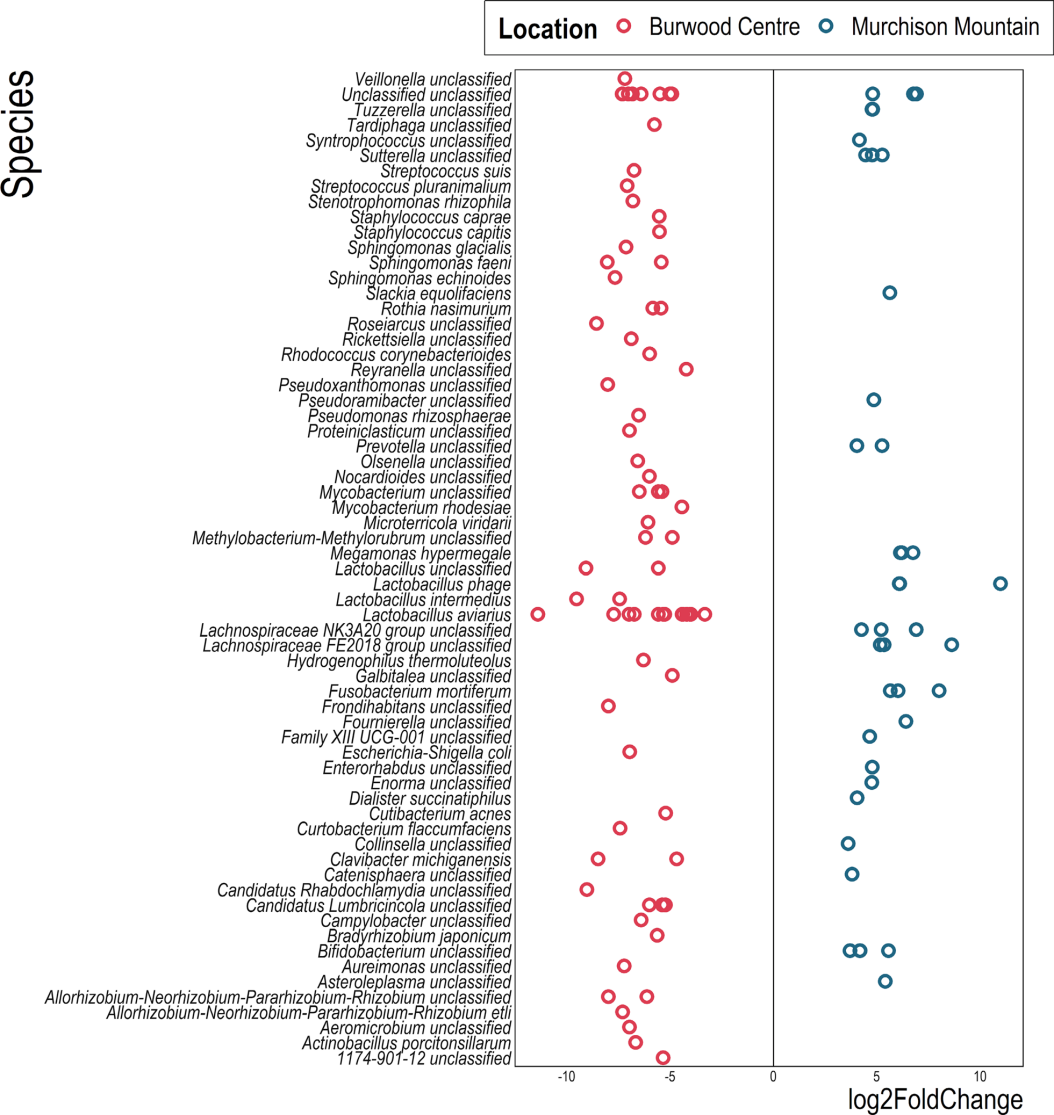
Differentially abundant ASVs (*p* value < 0.01; Wald test with local regression fit and Benjamini–Hochberg *p* value adjustment) between Burwood Takahē Centre and Murchison Mountain locations. Each circle represents an individual 16S rRNA gene defined ASV

Differential abundance analysis of ASVs between Burwood Takahē Centre and Te Puhi-a-Noa Murchison Mountain takahē samples identified 116 ASVs spanning 56 genera and 65 species that significantly differed in relative sequence abundance (*p* value < 0.01; Wald test with local regression fit and Benjamini–Hochberg adjustment) between the two locations (Fig. 4). ASV64_*Lactobacillus aviarius* had 11-fold higher abundance in captive Burwood Takahē Centre samples compared to wild Te Puhi-a-Noa Murchison Mountain samples, while core members ASV1_*Lactobacillus aviarius,* ASV16_*Lactobacillus aviarius,* ASV36_*Lactobacillus intermedius* had threefold, fourfold, and 9.5-fold greater relative sequence abundance, respectively, in Burwood Takahē Centre samples. Burwood Takahē Centre samples also hosted several strains of *Lactobacillus* that were at least fivefold higher in relative sequence abundance compared to Te Puhi-a-Noa Murchison Mountain samples. No *L. aviarius*, *L. intermedius*, or unclassified *Lactobacillus* ASVs were identified as significantly more abundant in the Te Puhi-a-Noa Murchison Mountain samples. However, ASV30_*Lactobacillus phage* displayed 11-fold higher relative sequence abundance in wild Te Puhi-a-Noa Murchison Mountain takahē samples, and core member ASV3_*Fusobacterium mortiferum* had sixfold greater relative sequence abundance.

## Discussion

The takahē is a Nationally Vulnerable [56] rail with < 450 birds distributed across various sanctuaries and centres throughout Aotearoa New Zealand. Intensive conservation strategies began in 1960 when amateur ornithologist Elwin Welch successfully raised four takahē chicks on his farm near Pūkaha [15]. The Takahē Recovery Programme now supports various scientific research projects to aid their strategies for improving takahē health and reproduction. With microbiota research becoming increasingly popular in the last decade, conservation biology is beginning to incorporate studies on the microbiota of threatened species in an effort to better understand animal biology and health [64, 74]. In Aotearoa New Zealand significant headway has been made in researching the gut microbiota of its only other flightless herbivorous avian species, the critically endangered kākāpō, where a low-diversity microbiota was an unexpected finding in this unique parrot [67, 69, 71]. This paper represents the first study to examine the bacterial microbiota of the takahē gastrointestinal tract and provides evidence for significant compositional differences in microbiota profiles among sampling locations.

### The takahē gut microbiota is diverse but frequently dominated by *Lactobacillus*

The takahē gut hosts a moderately diverse microbiota that appears to be frequently dominated by members of the *Bacteroidota*, *Firmicutes* and *Proteobacteria* phyla. The most abundant ASV was identified as a strain of *Lactobacillus aviarius araffinosis* which was present in all birds at an average relative sequence abundance of 17%. In fact, it was the only member of the microbiota to be detected in > 90% of the sampled population. *Lactobacillus* is a commonly described commensal in birds and has been suggested as a probiotic and alternative treatment to antibiotic administration in poultry [35]. *L. aviarius araffinosis* is a homofermentative bacterium that produces lactic acid from sugar metabolism, and we assume it plays a key role in breaking down plant material ingested by takahē. While *Lactobacillus* was present in all samples, its relative sequence abundance varied considerably. Location was a significant source of this variation, with samples from Burwood Takahē Centre hosting higher levels of *Lactobacillus* compared to other sites. Differences in supplementary feeding among sites would not appear to explain the dominance of *Lactobacillus* in some samples: all birds at Burwood Takahē Centre are supplementary fed yet not all faecal samples from Burwood were dominated by *Lactobacillus*. However, it likely contributes to the observed variation among bacterial communities. There was a significant difference in the Inverse Simpson diversity measure between samples from takahē which were regularly provided supplementary feed versus those that were not. Regularly fed takahē had lower Inverse Simpson scores, but again this overall significant difference is likely in relation to *Lactobacillus* dominance in some Burwood Takahē Centre takahē. The collection of more metadata and samples is necessary to tease apart the relationship between *Lactobacillus* abundance and the takahē gut microbiota.

Other core members of the takahē gut bacterial community identified at 70% prevalence included members of the two dominant phyla *Bacteroidota* (*Prevotella*) and *Firmicutes* (*Lactobacillus aviarius*, *Lactobacillus intermedius*, *Lactobacillus* sp., *Clostridium *sensu stricto* 1*, and *Solobacterium*), but also included *Bifidobacterium* (*Actinobacteriota*) and *Fusobacterium mortiferum* (*Fusobacteriota*). Interestingly there were no *Proteobacteria* ASVs identified as core members of the gut community despite this phylum constituting a large proportion of overall sequence reads. The relative sequence abundances of core genera observed in wild takahē was not markedly different from the patterns observed for insurance population samples. Te Puhi-a-Noa Murchison Mountain samples displayed similar relative sequence abundance of *Bifidobacterium*, *Campylobacter*, *Lactobacillus*, *Prevotella*, *Pseudomonas* and *Solobacterium* to samples from Cape Sanctuary, the Foveaux Strait island, and Mana Island where the habitat differs substantially from the tussock-dominated mountains and is mostly characterised by regenerating native forest and European grasses on formerly-grazed land. Anthropogenic management is reduced on the Foveaux Strait island, but for the population at Cape Sanctuary and Mana Island management practices remain similar to other insurance population sites and are considerably greater than the wild population which is largely undisturbed.

The commensal bacterium *Campylobacter* previously identified in takahē [22, 23] was reasonably prevalent across the current data set and identified as a core genus of the takahē gut microbiota. Grange et al. [23] reported that the prevalence of three *Campylobacter* species (*C.* sp. *nova 1*, *C. jejuni*, and *C. coli*) varied among subpopulations in relation to conservation management practices and presence of adjacent farmland. There was also location-associated differentiation of *Campylobacter* sp. *nova 1* genome sequence types [22]. In the current study we identified a strain of *Campylobacter jejuni* (ASV26) that was detected in 68% of faecal samples. Grange et al. [23] found *C. jejuni* to be significantly more prevalent in the wild population compared with insurance and captive populations; in the current study *C. jejuni* had 100% prevalence at all sites except Burwood Takahē Centre, Tiritiri Matangi Island and Motutapu Island, where the prevalence was 42.9%, 42.9% and 75%, respectively. These conflicting results likely arise from discrepancies between locations sampled in our study versus those of  Grange et al. [23], but the overall trend of wild takahē hosting greater prevalence of *C. jejuni* in comparison to the captive Burwood Takahē Centre population is supported by the current study.

### Location significantly affects composition of the takahē gut microbiota

Bacterial composition of the takahē gut varied significantly among sanctuaries, with location explaining 32% (21% after accounting for supplemental feeding) of the observed microbiota variation (Fig. 2). Burwood Takahē Centre bacterial communities stood out as having the lowest diversity compared to the other seven locations (Additional file 5: Figure S3). Samples from Burwood Takahē Centre also tended to cluster separately from other samples (Fig. 2), further emphasising the dissimilarity of their communities. This is at least partially driven by the dominance of *Lactobacillus* in some Burwood Takahē Centre samples, where this genus comprises up to 82% of obtained 16S rRNA gene sequences, and by the relative lack of *Prevotella* in the majority of Burwood Takahē Centre samples (though this could reflect the compositionality of our 16S rRNA gene sequence data [18, 44, 73]). Te Puhi-a-Noa Murchison Mountain samples appear to host significantly higher relative sequence abundances of ASVs from lineages relating to the fermentation of plant polysaccharides, including *Lachnospiraceae*, *Prevotella*, *Fusobacterium* and *Bifidobacterium*, possibly indicating greater fermentative potential in the wild takahē gut. Since wild takahē are not provided supplemental feed they may have a greater dependency on gut microbes capable of fermenting plant polysaccharides to obtain their daily energy and nutrient requirements. Te Puhi-a-Noa Murchison Mountain samples also hosted a greater relative sequence abundance of unclassified genera than samples from most other sites, particularly those from Burwood Centre. Supplementary feeding had a significant effect on bacterial community composition but was essentially nested inside location groupings, as evidenced by the centroid locations of 'regular' versus 'no' supplemental feed groups largely overlapping the centroids of Burwood Takahē Centre and Te Puhi-a-Noa Murchison Mountain groups, respectively. It is therefore unclear whether supplementary feeding is actually having a biological effect on the takahē gut microbiota or if some other variable related to these locations is responsible. Established versus regenerating habitat type was also significantly associated with gut microbiota variation, though it had a small effect size and was nested in location groupings. However, this result may only reflect uneven dispersion within the two groups given established habitat was present only at Murchison Mountain and Burwood Takahē Centre. Despite being geographically close, the Burwood Takahē Centre and Te Puhi-a-Noa Murchison Mountain populations, and Motutapu, Rotoroa and Tiritiri Matangi Island populations harbour substantially different gut bacterial communities. Though not directly tested in this study, similar climate conditions do not appear to result in similar gut microbiota profiles for samples from those corresponding sites.

### Implications for takahē conservation

A key motivation for this study was to determine whether aspects of the takahē gut microbiota could be used to inform the conservation and management of this enigmatic species. We were able to identify that takahē from Burwood Takahē Centre hosted far greater relative sequence abundance of *Lactobacillus* (and corresponding lack of *Prevotella* species) compared to less intensively managed sites. An obvious point of difference between the captive and wild populations is supplementary feeding with cereal-based pellets. While supplementary feed was identified as a significant factor influencing bacterial community composition, the results are somewhat confounded by the incidence of supplemental feeding at locations categorized by relative captive management in this study. In light of these initial results, we recommend further research should be undertaken to assess potential effects of the cereal pellets’ current composition on the takahē gut microbiota. The recent establishment of a second wild takahē population in Kahurangi National Park (and recent translocations to the Te Puhi-a-Noa Murchison Mountains) provides an excellent opportunity for such a study to investigate whether supplemental feed is indeed influencing composition of the gut microbiota by increasing sample size and diversity of locations with similar abiotic factors. Altered microbiotas in captivity may have significant impact on subsequent release and restocking efforts [74]. Identifying essential microbes that may be lacking in captive takahē could make good candidates for probiotic supplements to improve translocation success.

Despite captive takahē faecal samples hosting substantially different bacterial communities to those of the wild population, Burwood Takahē Centre takahē are the only external subpopulation in this study to have access to similar tussock habitat as observed for the Te Puhi-a-Noa Murchison Mountain population, albeit with reduced variety. We can only surmise that factors relating to captivity in Burwood Takahē Centre play a much larger role in shaping the gut microbiota than does access to similar wild resources. Captivity significantly alters mammalian gut microbiotas across taxonomically and ecologically diverse species [42]. While the takahē subpopulations are fragmented throughout the country, they are in fact fairly connected via regular translocation networks (described in [21]). As the predominant breeding centre, Burwood Takahē Centre represents the hub of this busy translocation network and thus provides ample opportunity for mixing of microbiotas from various habitat types spanning > 1100 km. Given the potential for a variety of microorganisms to be introduced to the breeding centre during translocation, one might expect to see Burwood Takahē Centre takahē hosting more diverse gut microbiota profiles. Yet Burwood Takahē Centre samples were the least diverse of all eight locations tested and were largely dominated by *Lactobacillus*, particularly ASV1 *Lactobacillus aviarius araffinosis*. Though speculative, the high abundance of *Lactobacillus* in some Burwood Takahē Centre takahē could potentially reflect immunologically naïve systems where *Lactobacillus* has thrived and dominated other members of the microbiota, especially in younger takahē. Similarly, more frequent medical treatment in a captive environment may also explain why one bacterium tends to dominate the community. Antibiotic treatment can profoundly change both the composition and function of the gut microbiota, and (at least in humans) can even lead to long-lasting effects including the development of autoimmune diseases such as allergies and inflammatory bowel disease [4, 57, 82]. Testing the effect of medical treatment on the takahē gut microbiota is thus another logical future step to better our understanding of external factors that influence gut community composition and function. Shotgun metagenome sequencing would greatly enhance our current knowledge of the gut bacterial community to include not only functional information, but also give valuable insight into the fungal, archaeal, and viral components of the takahē microbiome (defined as the microbiota plus their theatre of activity [7, 38]). Obtaining functional information is essential to the conservation aspect of future projects to understand if altered microbial communities are losing important metabolic functions [2, 9, 71], or whether variable gut microbiomes perform similar activities regardless of community membership.

The microbiome plays an essential role in development and training of the host immune system [82], notwithstanding its critical role in other aspects of host health (i.e. neurological, digestive, and reproductive systems [17, 41]). This complex ecosystem of microorganisms is, however, susceptible to external perturbation. The role of anthropogenic disturbance, such as providing medical treatment to threatened species, may have unforeseen consequences on host animal immunity, health, and fitness in relation to altered gut microbial communities [64, 74]. Conceivably, variation in the takahē gut microbiota could in some instances reflect variable immunological function among sanctuaries. In sites where immunity may be reduced with altered gut microbiota, takahē could be more susceptible to gut pathogens such as coccidia, *Salmonella*, enteropathogenic *Escherichia coli* or *Erysipelothrix rhusiopathiae*, inevitably transmitting these diseases to other subpopulations via translocation. Parasitic larvae are known to alter gut microbial diversity and reduce levels of circulating antibodies in birds [32], leaving them susceptible to further secondary infections. Takahē subpopulations are thought to differ in their tendency to harbour and transmit infectious organisms, based on variable carriage of *C. coli* and *C. jejuni* [23], which could be attributable to altered composition of the gut microbiota. Advancing our knowledge of how the takahē gut microbiome responds and interacts with pathogenic microorganisms can only help to further refine conservation management practices, especially with regard to potential pathogen transmission with translocation.

Our study of the takahē gut microbiota is limited by the absence of a typical control population, as is often the case when working with threatened species. Though we have used samples from the wild Te Puhi-a-Noa Murchison Mountain population to make comparisons with more intensively managed subpopulation microbiota profiles, the takahē rediscovered here only survived European colonisation and subsequent mammalian predation due to the remoteness and harsh climate of this area [12, 28, 29, 40]. The population has also been heavily supplemented over time by birds reared at the Takahē Burwood Centre. It is unlikely the current site represents ideal takahē habitat given they were once widespread across ecologically diverse habitats in both Te Ika-a-Maui North and Te Waipounamu South Islands of Aotearoa New Zealand [3, 65]. Historically, the takahē gut microbiota may have varied geographically and been functionally flexible depending on available resources in different habitat types. Perhaps the variation of bacterial community composition by location in our current study reflects this speculative hypothesis that takahē have some degree of flexibility in the interactions between microbiota, location, and diet. If so, it would be a promising sign for the conservation management of this unique endemic species.

### Concluding remarks

This study presents a first step towards understanding the role of the gut microbiota in takahē biology and health. Overall, gut microbiota diversity differed among locations and captive individuals hosted much greater relative abundance of *Lactobacillus* compared to takahē sampled at other sites. We have outlined areas that require further investigation, particularly regarding the collection of extra material and metadata to help facilitate comparisons between gut microbiota samples and perhaps elucidate some of the significant variation between locations observed in this study. This information will help inform the conservation and management of our nationally threatened takahē, and safeguard their survival for future generations.

## Supplementary Information

**Additional file 1:** Metadata collected or available for takahē faecal samples analysed in this study.

**Additional file 2:** Taxonomic assignments of amplicon sequence variants (ASVs) obtained from takahē faecal samples.

**Additional file 3:** Non-rarefied ASV table.

**Additional file 4:** R markdown file used for statistical analyses of ASV data.

**Additional file 5:** Supplementary tables and figures.

## Data Availability

The raw sequence data are available in the NCBI SRA repository, under Bioproject accession number PRJNA737580. Metadata, taxonomic assignments, the non-rarefied ASV table and RStudio data analysis have been included as Additional files 1, 2, 3 and 4 respectively.
