# Supplementary material for: Gut microbiota of the threatened takahē: biogeographic patterns and conservation implications
Source: Anim Microbiome. 2022 Jan 25;4:11. doi: 10.1186/s42523-021-00158-5 (PMC8790836; doi:10.1186/s42523-021-00158-5)
Supplement: Supplementary file 5 — Additional file 5: Supplementary tables and figures. [file 42523_2021_158_MOESM5_ESM.docx]

Supplemental Table 1: Statistical outputs for pairwise PERMANOVA comparisons of significant models identified in Table 1. p-values are Benjamini-Hochberg adjusted for multiple comparisons. Significant p-values are denoted with asterisks (p < 0.05 = *, p < 0.01 = **, p < 0.001 = ***). B.C. = Burwood Takahē Centre, C.S. = Cape Sanctuary, F.S.I. = Foveaux Strait island, M.I. = Mana Island, M.T.I. = Motutapu Island, M.M. = Te Puhi-a-Noa Murchison Mountains, R.I. = Rotoroa Island, and T.M.I. = Tiritiri Matangi Island.

|  | Pairwise PERMANOVA for Bray-Curtis matrix | | | Pairwise PERMANOVA for gUniFrac matrix | | |
| --- | --- | --- | --- | --- | --- | --- |
| Covariate | p - value | F | R^2^ | p - value | F | R2 |
| Location | | | | | | |
| B.C. vs C.S. | 0.01* | 5.30 | 0.25 | 0.02* | 6.79 | 0.30 |
| B.C. vs F.S.I. | 0.006** | 5.58 | 0.25 | 0.02* | 5.49 | 0.24 |
| B.C. vs M.I. | 0.006** | 5.02 | 0.19 | 0.03* | 3.63 | 0.01 |
| B.C. vs MT.I. | 0.006** | 4.68 | 0.23 | 0.02* | 3.57 | 0.18 |
| B.C. vs M.M. | 0.006** | 8.55 | 0.30 | 0.02* | 8.55 | 0.30 |
| B.C. vs R.I. | 0.03* | 3.02 | 0.15 | 0.03* | 2.87 | 0.14 |
| B.C. vs T.M.I. | 0.006** | 4.25 | 0.18 | 0.04* | 2.42 | 0.11 |
| C.S. vs F.S.I. | 0.09 | 1.73 | 0.20 | 0.14 | 1.33 | 0.16 |
| C.S. vs M.I. | 0.09 | 1.79 | 0.13 | 0.09 | 2.15 | 0.15 |
| C.S. vs MT.I. | 0.04* | 2.33 | 0.28 | 0.05 | 6.18 | 0.51 |
| C.S. vs M.M. | 0.02* | 2.87 | 0.22 | 0.02* | 2.60 | 0.21 |
| C.S. vs R.I. | 0.03* | 2.36 | 0.25 | 0.03* | 3.98 | 0.36 |
| C.S. vs T.M.I. | 0.08 | 1.82 | 0.17 | 0.08 | 3.33 | 0.27 |
| F.S.I. vs M.I. | 0.07 | 1.97 | 0.13 | 0.09 | 1.95 | 0.13 |
| F.S.I. vs MT.I. | 0.03* | 2.91 | 0.29 | 0.05 | 3.83 | 0.35 |
| F.S.I. vs M.M. | 0.01* | 2.97 | 0.21 | 0.03* | 2.65 | 0.19 |
| F.S.I. vs R.I. | 0.03* | 2.34 | 0.23 | 0.08 | 2.40 | 0.23 |

| F.S.I. vs T.M.I. | 0.05 | 2.09 | 0.17 | 0.10 | 2.26 | 0.19 |
| --- | --- | --- | --- | --- | --- | --- |
| M.I. vs MT.I. | 0.01* | 2.48 | 0.17 | 0.05 | 2.58 | 0.18 |
| M.I. vs M.M. | 0.04* | 2.76 | 0.15 | 0.05 | 2.60 | 0.14 |
| M.I vs R.I. | 0.07 | 1.96 | 0.13 | 0.12 | 1.69 | 0.12 |
| M.I. vs T.M.I. | 0.07 | 1.95 | 0.12 | 0.20 | 1.34 | 0.08 |
| MT.I. vs M.M. | 0.009** | 5.32 | 0.35 | 0.02* | 8.12 | 0.45 |
| MT.I. vs R.I. | 0.09 | 1.64 | 0.19 | 0.09 | 2.00 | 0.22 |
| MT.I. vs T.M.I. | 0.32 | 1.12 | 0.11 | 0.26 | 1.27 | 0.12 |
| M.M. vs R.I. | 0.01* | 4.17 | 0.28 | 0.02* | 4.96 | 0.31 |
| M.M. vs T.M.I. | 0.01* | 3.82 | 0.23 | 0.03* | 4.32 | 0.25 |
| R.I. vs T.M.I. | 0.16 | 1.43 | 0.13 | 0.26 | 1.21 | 0.11 |
| Supplemental feeding | | | | | | |
| Regular vs None | 0.003** | 4.03 | 0.07 | 0.005** | 4.71 | 0.09 |
| Regular vs Occasional | 0.005** | 2.28 | 0.05 | 0.02* | 2.37 | 0.05 |
| None vs Occasional | 0.003** | 4.76 | 0.24 | 0.003** | 6.33 | 0.30 |


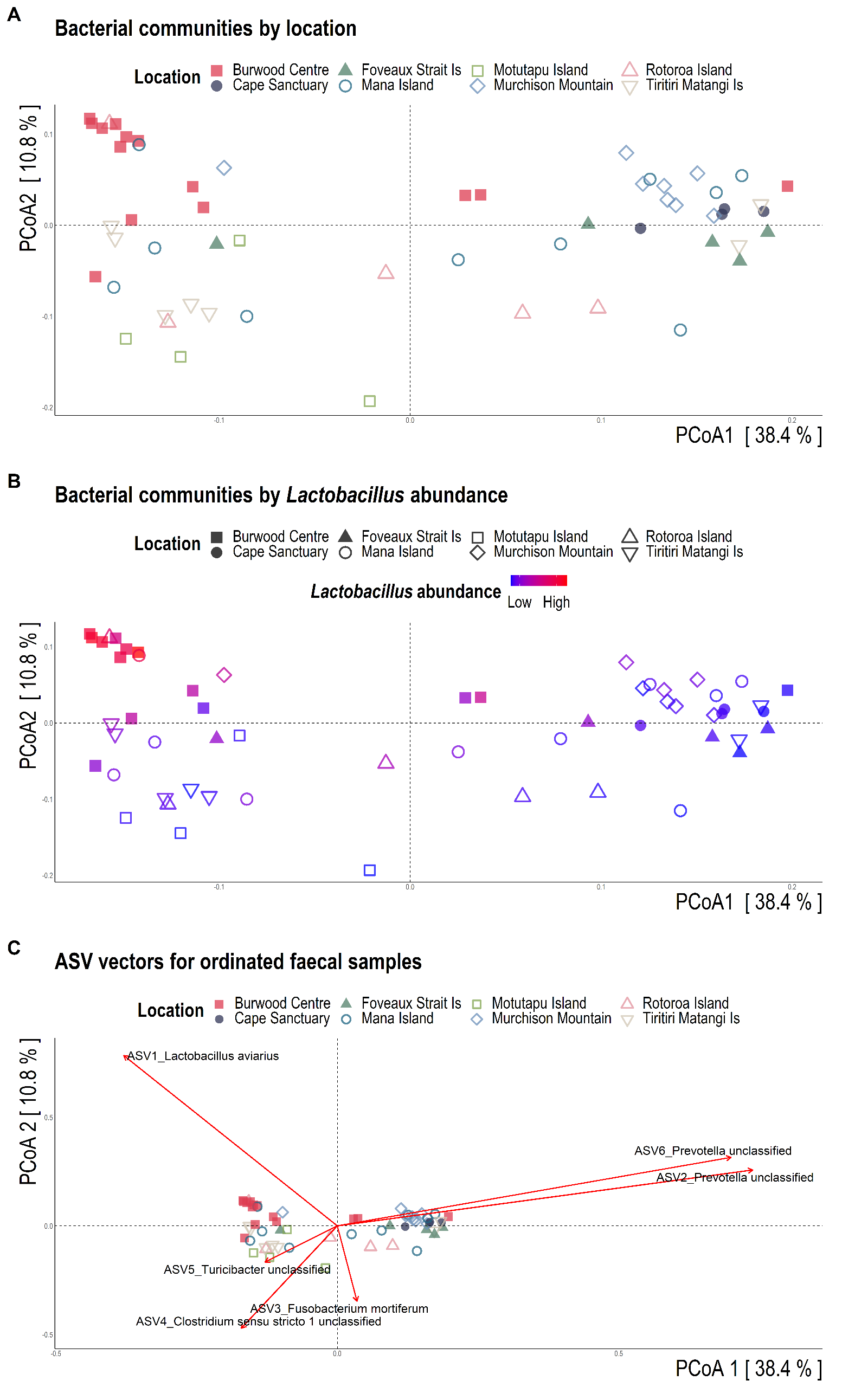


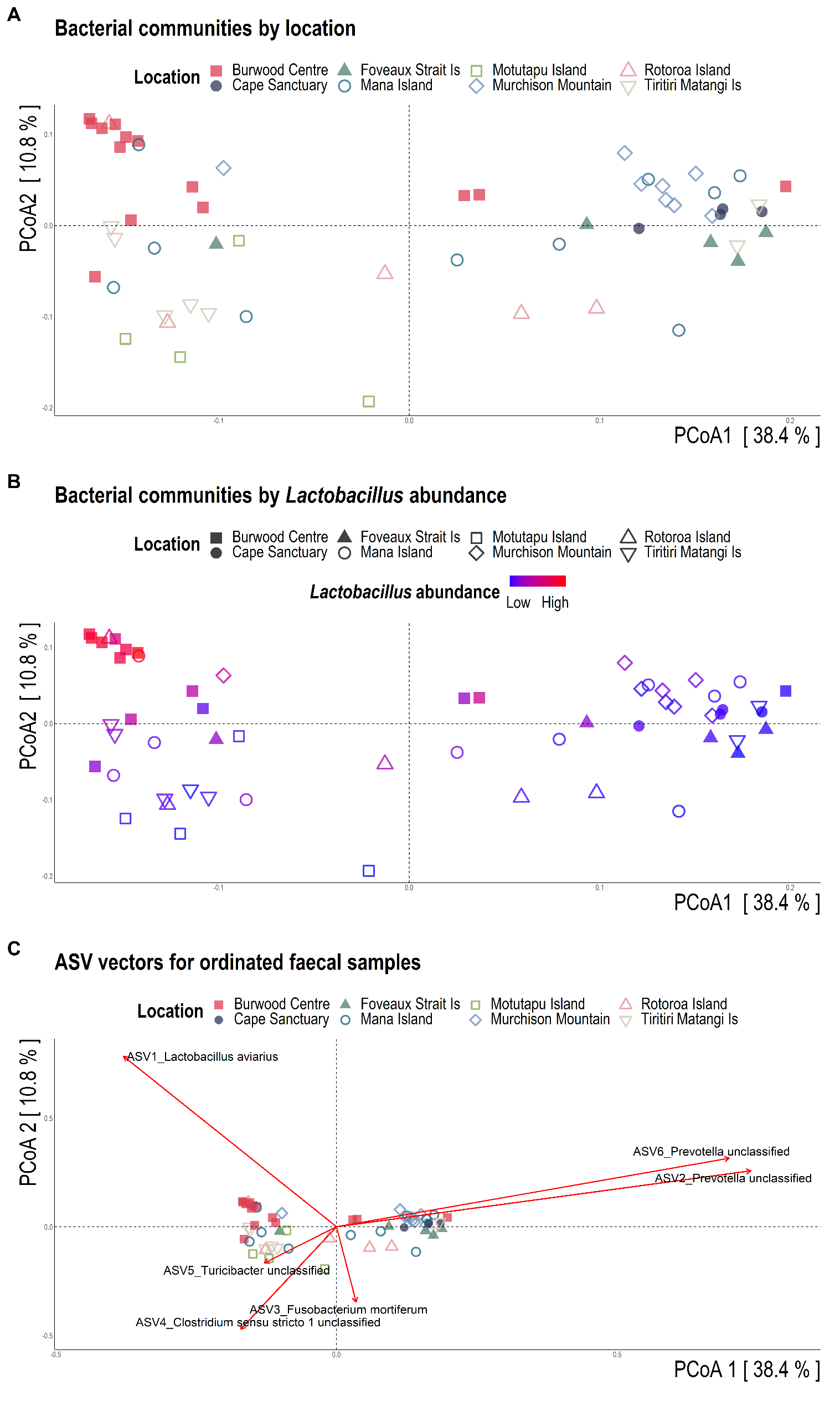


***Supplemental Figure 1*** *16S rRNA gene sequence-based generalised GUniFrac dissimilarity distances visualised via principal coordinate multidimensional scaling ordination. Each dot of the PCoA represents the microbiota of a single takahē faecal sample. [A] bacterial communities coloured and shaped by location. [B] bacterial communities coloured by relative* Lactobacillus *abundance with location groupings as different shapes. [C] bacterial communities coloured and shaped by location with the 6 most abundant ASVs plotted as vectors using the vegan::envfit function.*

*
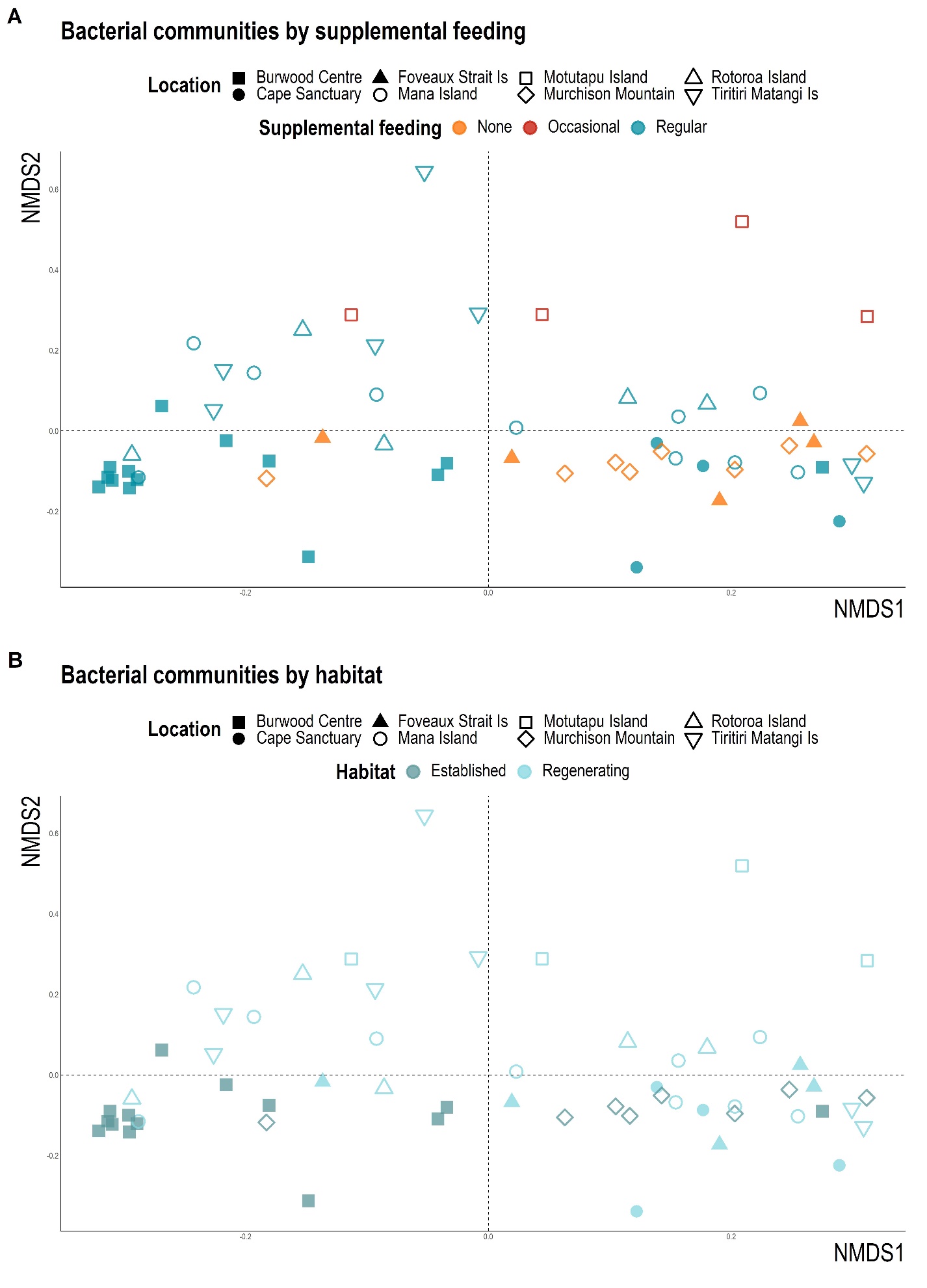
*

***Supplemental Figure 2*** *16S rRNA gene sequence-based Bray-Curtis dissimilarity distances visualised via non-metric multi-dimensional scaling ordination. [A] coloured by supplemental feeding status and shaped by location. [B] coloured by habitat type and shaped by location. Each dot of the NMDS represents the microbiota of a single takahē faecal sample (MDS stress = 0.17).*





***Supplemental Figure 3*** *Alpha-diversity measures grouped by [A] location [B] supplemental feeding, and [C] habitat type. Benjamini–Hochberg adjusted p-values are denoted for comparisons that differed significantly. Significant comparisons are denoted by asterisks (p < 0.05 = *, p < 0.01 = **, p < 0.001 = ***).*
